# Fluid Resuscitation in Malaria: The Need for New Randomised Clinical Trials

**DOI:** 10.1371/journal.pctr.0010024

**Published:** 2006-09-15

**Authors:** Nick Day

## Background

Very little clinical research has been conducted on how to manage children with severe malaria, despite it accounting for the deaths of around 1 million children each year in Africa alone.

Antimalarial drugs remain the mainstay of treatment (and are the subject of several current or planned clinical trials), but there is increasing debate around aspects of supportive care and the treatment of complications. No aspect of care has been more controversial in recent years than the initial fluid management of children with severe malaria, particularly given the resource-poor health-care context in which most such children are treated. The debate centres around the role of hypovolaemia (i.e., insufficient circulating blood volume) in the pathophysiology of severe malaria. Hypovolaemia has been incriminated as an important cause of metabolic acidosis, which has been shown repeatedly to be associated with a poor prognosis. The key question is whether hypovolaemia contributes to impaired tissue perfusion in severe malaria, contributing to the anaerobic glycolysis and consequent acidosis. Some studies of severe malaria in children have supported this hypothesis by providing indirect evidence (e.g., capillary refill time, central venous pressure measurements, raised creatinine, and clinical dehydration) that severely ill patients are commonly hypovolaemic on admission, and that this contributes to the severity of the disease [1,2]. In febrile children hypovolaemia often results from dehydration, but a detailed study in Gabon showed that the children with severe malaria investigated had only a mild or moderate degree of dehydration (as measured by total body water, not necessarily synonymous with hypovolaemia) [3]. So, should children with severe malaria all receive rapid intravenous fluid rehydration? And if so, with what fluid?

Proponents of rapid fluid repletion cite the standards of care applied in resource-rich settings for severely ill children with bacterial sepsis, whilst those advocating caution argue that malaria should be considered differently, particularly given the haemodynamic and circulatory differences from sepsis and the concerns of precipitating or worsening pulmonary or cerebral oedema [2,4].

It is clear that clinical trials are the only way forward to resolve the debate. There have been several previously published intervention studies, all conducted by Maitland and the Kilifi team on the Kenyan coast, but to date all have been too small and heterogenous to provide conclusive answers.

Should children with severe malaria all receive rapid intravenous fluid rehydration? And if so, with what fluid?

The largest of these studies did provide, in a sub-group analysis of severely acidotic patients, a tantalising suggestion of a mortality benefit from fluid resuscitation with albumin 4.5%, when compared with saline. But there was no “maintenance fluids only” control group, and as mortality was relatively high in the saline group, a deleterious effect of saline bolus could not be excluded. There is, however, a scientific rationale suggesting that albumin might possibly be beneficial in severe malaria, as it has been shown to have a number of physiological effects other than on colloid oncotic pressure. In particular, there is recent evidence that albumin may be neuroprotective in acute ischemic stroke [5].

## The Study's Key Findings

Akech et al. have conducted a phase II study [6] examining the safety and efficacy of fluid resuscitation with Gelofusine (a gelatin-based synthetic colloid) and with albumin in 88 children with severe malaria and metabolic acidosis but without severe anaemia. Sixty percent of the patients had cerebral malaria. In terms of safety it was reassuring that no patients developed pulmonary oedema, and only two developed clinical signs of raised intracranial pressure (both in the Gelofusine group).

Though there was no difference between the two groups in terms of the primary outcomes, resolution of shock and of acidosis, there was a suggestion on analysis of the secondary outcomes of a mortality benefit in favour of albumin (*p* = 0.06). In addition, the authors pool the data from all their trials comparing albumin with other resuscitation fluids, and find that albumin administration is associated with a very significant overall mortality benefit.

## Limitations of This Study

In this phase II study, patients were not individually randomised but rather allocated to the treatments in blocks of ten, though it appears from the baseline data that the resulting two groups were evenly matched. This has no implications for the safety data, but puts limitations on the interpretation of the mortality data. In addition, mortality was not a primary endpoint for either this study or any of the previous studies cited in the pooled analysis, so the finding of a mortality benefit from albumin administration should be regarded as hypothesis-generating rather than definitive, despite the impressive *p-*value (*p* = 0.004). Although the safety data are reassuring, the lack of a “maintenance fluids only” control group limits the study's impact on the overall debate over whether aggressive fluid resuscitation should be given at all.

## Implications for Future Research

Give that severe malaria is such an important disease in terms of morbidity and mortality throughout the tropical world, it is astonishing how few clinical trials have addressed its treatment. This is particularly true for the non-antimalarial-drug aspects of management; few trials have been carried out, and those that exist have often been inadequately powered and of poor quality [7]. No adjunctive therapy has ever been shown to be beneficial. Akech and colleagues are to be congratulated for pursuing practical clinical answers to how the management of severe malaria in Africa can be improved. Out of their work and that of others in the field, including those who oppose aggressive fluid resuscitation, two separate but interconnected clinical questions arise. Firstly, is aggressive fluid therapy in severe childhood malaria indicated? Secondly, does fluid resuscitation with albumin have specific benefits, such as a neuroprotective effect in comatose patients? Only large, adequately powered, probably multi-centre, randomised clinical trials can answer these questions. Surrogate markers, such as resolution of acidosis (despite its central role in the original hypothesis), have not been proven to be linked with mortality. The current study provides safety data to support a large trial of fluid resuscitation, as well as providing hypothesis-generating data to support inclusion of albumin as an arm in such a trial. Although albumin is prohibitively expensive by African health-care standards (about US$35 per treatment), with the planned widespread use of long-term antiretroviral therapy in Africa the accepted precedents are changing, and cost should not be a reason to exclude albumin from assessment. Any study must have a “maintenance fluids only” control arm, as the risk–benefit ratio of fluid resuscitation per se, particularly with saline, remains unclear. The debate on fluid management is a healthy one, and the few groups working in this field should work together to produce a clinical trial design that will answer these important questions. The potential benefits for African children with severe malaria are enormous. 
